# Clinically significant endoscopic findings in a dyspeptic rural population cohort of Sindh, Pakistan: Are we over-investigating?

**DOI:** 10.12669/pjms.38.6.5948

**Published:** 2022

**Authors:** Muhammad Kamran, Asher Fawwad, Bushra Rabbani, Jameel Ahmed

**Affiliations:** 1 Dr. Muhammad Kamran, FCPS Department of Gastroenterology, Fazaia Ruth Pfau Medical College, Karachi, Pakistan; 2 Dr. Asher Fawwad, PhD Professor & Head of the Biochemistry Department, Baqai Medical University, Karachi-Pakistan. Research Director (Honorary), Department of Research, Baqai Institute of Diabetology and Endocrinology, Baqai Medical University, Karachi-Pakistan; 3 Dr. Bushra Rabbani, FCPS, Department of Medicine, Fatima Hospital, Baqai Medical University, Karachi-Pakistan; 4 Dr. Jameel Ahmed, FRCP Department of Medicine, Fatima Hospital, Baqai Medical University, Karachi-Pakistan

**Keywords:** Upper gastrointestinal endoscopy, Dyspepsia, Gastritis, Findings

## Abstract

**Objectives:**

To discuss the clinically significant endoscopic findings of the upper GI tract and their association with different age groups in a dyspeptic rural population of Gadap town, Sindh.

**Methods:**

This was a retrospective secondary data analysis of 806 patients conducted in the medical ward of Fatima Hospital, Baqai Medical University from December 2016 to May 2019. It was approved by the University Ethics Committee. Patients’ demographics and other data related to the procedure were recovered from patients’ records. SPSS version 20 was used for statistical analysis.

**Results:**

There were marginally more women suffering from dyspepsia as opposed to men (51.5% vs 48.5% respectively). Majority of the patients were less than 45 years of age, with most procedures being performed as outpatients. Nearly 40% of the patients did not have clinically relevant endoscopic findings. Most common significant finding on endoscopic examination was gastritis followed by hiatal hernia.

**Conclusion:**

Normal upper gastrointestinal endoscopy, regardless of age, is a common finding in patients suffering from dyspepsia in our setting. Therefore, in a resource constraint environment like ours, expensive endoscopic procedures should be reserved for those patients who are not responding to medical therapy or those who have alarm symptoms.

## INTRODUCTION

Dyspepsia can be clinically defined as epigastric pain which lasts for at least four weeks, accompanied by a feeling of fullness in the stomach, nausea, vomiting or heart-burn.^1^ Many a times, these patients harbor clinically significant pathologies in their gastrointestinal (GI) tract for e.g. gastritis, peptic ulcers and various types of cancers. However, when a patient with dyspeptic symptoms undergoes an upper gastrointestinal endoscopy (UGIE) which rules out any organic pathology, then such individuals are said to be suffering from “functional” dyspepsia.^1^ Based on a recent meta-analysis, dyspepsia occurs in at least one-fifth of the population in general.^2^ However, its prevalence can be as high as 50% in some Asian countries.^3^ Although dyspepsia is not a life-threatening condition and doesn’t affect survival, it has a significant impact on the quality of life of an individual and also increases healthcare costs by frequent physician visits and polypharmacy.^4^

Evaluation of dyspepsia can be performed by various means. These include therapeutic (empiric) trials of medications which neutralize or suppress gastric acid production, non-invasive testing for helicobacter pylori (a common micro-organism responsible for producing dyspeptic symptoms) and invasive techniques like UGIE.^5^ Among these, the “gold standard” remains to be UGIE, as it permits direct visibility of the inner lining of the GI tract and hence perform biopsies as well as intervene in emergency situations like upper GI bleed.^6^ This procedure, however, is a costly one and affordability always remains an issue in a third world country like Pakistan, where people are already striving hard to make both ends meet. Furthermore, there are conflicting data regarding the utility of UGIE in diagnosing clinical significant lesions in patients with dyspepsia.^7^ Also, there is an on-going debate as to whether or not these procedures are being performed for appropriate indications, keeping in view the relevant clinical practice guidelines.^8^

Gadap town, an area situated in the northern part of Karachi city, is unique in the sense that it comprises multi-ethnic groups, mostly having a poor socio-economic background. A significant population of Gadap town also suffers from dyspeptic symptoms. Therefore, the aim of this study was to look at the clinically significant findings of the upper GI tract based on endoscopy, in this distinctive rural cohort of individuals suffering from dyspepsia. Additionally, we also analyzed the correlation of these positive findings with age of the patients.

## METHODS

This study was conducted as a secondary data analysis of our previous work which has already been published. In that retrospective study, the common clinical indications, key endoscopic findings and their correlation with alarm symptoms were assessed in patients who underwent UGIE at Fatima Hospital, Baqai Medical University, from December 2016 to May 2019.^9^ The study was approved by the university ethics committee (Ref: BMU-EC/07-2019-03, Dated: July 19th 2019). All adult patients > 18 years of age were included in the analysis who had symptoms of dyspepsia. Diagnosis of dyspepsia was made if the patient complained of epigastric pain lasting for four weeks, accompanied by either of the following symptoms: feeling of fullness in the stomach, nausea, vomiting or heart-burn^1^. Patients < 18 years, and those who underwent UGIE for reasons other than dyspepsia (e.g. unexplained anemia, dysphagia, screening or surveillance for esophageal or gastric varices, and upper gastrointestinal bleeding) were excluded.

Out of the 1288 patients who went through the procedure, 806 (approximately two-thirds of the individuals) had symptoms of dyspepsia.^9^ Olympus Q140 series forward viewing video endoscope was used to carry out all procedures, with an overnight fast and in the standard manner after obtaining written informed consent. 4% xylocaine was used to administer pharyngeal analgesia. The patient population consisted of either those already admitted in wards, or as an out-patient setting (with either referrals from the out-patients department of Fatima Hospital or from private clinics within close proximity of the hospital). Before being discharged from the endoscopy unit, patients were kept in observation for approximately one hour to make sure they had no immediate post-procedure complications.

Information retrieved from the patients’ records included essential patient characteristics like age, gender, ethnicity and body mass index (BMI), along with other key informations like procedure setting (either inpatient or outpatient) and endoscopic findings. Abnormal endoscopy was considered as one in which there were macroscopic luminal findings. Biopsies were routinely taken from the lesions for histopathological examination. A cut-off age of 45 years was taken in order to differentiate younger patient population from the older one.^10^

Data entry and analysis was performed using statistical package for social sciences (SPSS) version 20, with descriptive variables being described as frequencies and percentages or mean± standard deviation. Two population proportion test was used to compare the endoscopic finding between age groups ≤45 years and >45 years. A P-value ≤0.05 was set as statistically significant.

## RESULTS

The characteristics of the study population are shown in Table-I. A total of 806 dyspeptic patients underwent UGIE during the said period, with slight female predominance. (51.5% females and 48.5% males). BMI was found similar in both genders (19.76±2.38 in men and 19.96±2.94 in women). Majority of patients were upto 45 years of age, whereas approximately 21% of the individuals were above 45 years. Almost half of the patients were of Pushto ethnicity. Most of the patients underwent the procedure as outpatients (90.6%). Clinically significant findings on UGIE in patients with dyspepsia are depicted in Table-II. More than two thirds of the patients (approximately 40%) had a macroscopically normal examination. However, prominent gross pathologic findings included gastritis (40.9%) and hiatal hernia (13.4%). The comparison of positive and clinically significant endoscopic findings between the younger and the older age groups are shown in Table-III. None of the p-values were significant, which means that in our study population, clinically significant findings do not associate with advance age group.

**Table I:** Characteristics of patients with dyspepsia undergoing upper GI endoscopy.

Variables	Number of patients (%)
N	806
***Age (years)***	
<30	353 (43.8)
31-45	285 (35.4)
46-60	129 (16.0)
61-75	34 (4.2)
>75	5 (0.6)
***Gender***	
Male	391 (48.5)
Female	415 (51.5)
***Ethnicity***	
Sindhi	298 (37.0)
Pushto	415 (51.5)
Urdu speaking	72 (8.9)
Punjabi	7 (0.9)
Balochi	13 (1.6)
Brohi	1 (0.1)
BMI*	
Male	19.76±2.38
Female	19.96±2.94
***Procedure setting***	
Out-patient	730 (90.6)
In-patient	76 (9.4)

* presented as mean±SD.

**Table II:** Predominant endoscopic findings in dyspeptic patients.

Endoscopic findings	Number of patients (%)
n	806
Normal examination	322 (39.9)
Gastritis	330 (40.9)
Candidia esophagitis	2 (0.25)
Non-candida esophagitis (including Barrett’s)	14 (1.7)
Hiatal hernia	108 (13.4)
Patulous GE junction	3 (0.3)
Duodenal fissuring	4 (0.49)
Esophageal/ gastric varices	5 (0.62)
Esophageal growth	3 (0.3)
Esophageal ulcer	1 (0.1)
Gastric growth	4 (0.49)
Gastric ulcer	2 (0.25)
Duodenal growth	3 (0.3)
Esophageal/ duodenal diverticulae	2 (0.25)
Achalasia	1 (0.1)
Worm infestation	2 (0.25)

**Table III:** Comparison of significant endoscopic findings with regards to age.

Endoscopic findings	Age≤45 years	Age>45 years	P-value	Overall
n	638	168	-	806
Non-candida esophagitis	11(1.7%)	3(1.8%)	N/A	14(1.7%)
Achalasia	0(0%)	1(0.6%)	N/A	1(0.1%)
Candida esophagitis	1(0.2%)	1(0.6%)	N/A	2(0.2%)
Duodenal fissuring	4(0.6%)	0(0%)	N/A	4(0.5%)
Duodenal growth	1(0.2%)	2(1.2%)	N/A	3(0.4%)
Esophageal growth	2(0.3%)	1(0.6%)	N/A	3(0.4%)
Esophageal ulcer	0(0%)	1(0.6%)	N/A	1(0.1%)
Esophageal/ duodenal diverticulum	2(0.3%)	0(0%)	N/A	2(0.2%)
Esophageal/ gastric varices	3(0.5%)	2(1.2%)	N/A	5(0.6%)
Gastric growth	4(0.6%)	0(0%)	N/A	4(0.5%)
Gastric ulcer	1(0.2%)	1(0.6%)	N/A	2(0.2%)
Gastritis	256(40.1%)	74(44%)	0.36	330(40.9%)
Hiatal hernia	86(13.5%)	22(13.1%)	0.892	108(13.4%)
Normal examination	263(41.2%)	59(35.1%)	0.15	322(40%)
Patulous GE junction	2(0.3%)	1(0.6%)	N/A	3(0.4%)
Worm infestation	2(0.3%)	0(0%)	N/A	2(0.2%)

Data presented as n (%); N/A: not applicable; P<value ≤0.05 considered to be statistically significant.

## DISCUSSION

Patients with dyspeptic symptoms are very commonly seen by both general physicians and gastroenterologists alike in their daily clinical practice. UGIE can easily discriminate between functional dyspepsia and symptoms caused by organic pathologies like esophagitis, peptic ulcer and cancers of the upper GI tract. Dyspepsia can severely impair the health-related quality of life of many individuals, and can also have significant negative effects on mental well-being. There is a strong association between dyspepsia and neuropsychiatric disorders like major depression.^11^

In our setting, more than two thirds of the patient population who presented with dyspeptic symptoms was young (< 45 years of age, with majority being < 30 years). A recent study conducted in a large teaching hospital in Iraq also revealed similar findings, with most of their dyspeptic population being under 55 years of age.^12^ Another older yet vital study from the United States reported the median age of dyspeptic patients to be 37 years.^13^ The reason for this symptom presentation at an early age is not very well understood, however it could be due to masking of abdominal symptoms with advancing age.^14^

Our study reported a slightly higher number of women with dyspepsia as compared to men. These findings concurred with the results of a Cambodian study which quoted a 54% female preponderance.^15^ Higher prevalence of dyspepsia in women has also been shown in a recent meta-analysis.^16^ The reason for this gender difference could be due to psychological influences, as well as certain hormonal factors leading to delayed gastric emptying in females.^17^ Since dyspepsia is mostly managed in an out-patient setting, majority (90%) of the endoscopic procedures were performed on out-patient population. This included those who were referred from the out-patients’ department of our hospital, as well as from general physicians practicing in the periphery.

We found that approximately 40% of the individuals undergoing UGIE for dyspeptic symptoms had no gross pathology in their upper GI tract. Data from local and regional studies published in the recent past have reported a higher percentage of normal endoscopic findings.^18^,^19^ However, we believe that our observation might be more accurate in this regard as the number of patients included in these studies were less as compared to our study. In general, this higher proportion of normal findings on endoscopy can be explained with the fact that most patients have access to acid suppressant drugs including proton pump inhibitors (PPIs) which are freely available over the counter. Another frequent finding, similar to the observations of a recent study from Cambodia, was the presence of gastritis, present in 40% of the patients.^15^

While comparing positive endoscopic findings with regards to age groups, we found none of the clinical findings to be statistically significant. This observation was in contrast to previously published data, where gross endoscopic pathologies were more commonly found in older individuals as opposed to younger ones.^15^,^20^ One reason for this discrepancy could be the fact that the population over 45 years was only one fifth of the total population that underwent UGIE in our study, and hence may not signify the true burden of diseases of the upper GI tract. Having said that, our cohort represents a real life scenario where younger people seek medical assistance more often than older ones due to the fear of having serious illness.

The American Society of Gastroenterology (ASGE) has categorically defined the appropriate indications for performing an UGIE^21^. However, unjustified and excessive utilization of an important investigative tool like UGIE is becoming an emerging concern at a global level.^21^ Numerous studies have shown that younger dyspeptic patients are less likely to benefit from UGIE as compared to older ones as far disease diagnosis is concerned. Despite this, many physicians and gastroenterologists use this examination as a tool for tailoring therapy for their dyspeptic patient population, even in the absence of alarm symptoms. The reasons for adopting this approach can be more than one. Firstly, gastric cancer is relatively common in people of low social class, and our study was also performed on patients belonging to an under-privileged society.^22^ Secondly, many patients with dyspepsia have an inner fear that they may be harboring serious GI related disorder, and an endoscopy which is negative for any such findings is a source of satisfaction for them.

### Strength and Limitations

The major strength of our study was that we were able to capture and present a comprehensive data including demographics, symptoms, and endoscopic findings of a fairly large multi-ethnic rural cohort suffering from dyspepsia. Originating from a tertiary care hospital serving the rural population of Sind, our data has its importance as it may help relevant healthcare authorities in formulating local practice guidelines for dyspeptic patients.

### Limitations of the study

Firstly, it was a retrospective analysis and therefore we relied on patients’ records, where important information may be missing for e.g. use of PPI prior to endoscopy. Secondly, this data was from a setup where cost is a major concern, so endoscopic biopsies were not performed in all dyspeptic patients who underwent the procedure. Lastly, being a single center hospital-based data, the findings may not represent the actual characteristics and behavior of diseases of the upper GI tract in this rural population. Large scale epidemiological studies are needed in this regard so that the true burden of such diseases can be estimated and dealt with in an effective manner.

## CONCLUSION

Dyspepsia is a universal disorder, and upper GI endoscopy is the safest and the most effective way to rule out sinister pathologies in patients suffering from dyspepsia. However, since a large number of patients (and even those > 45 years of age) in our cohort did not reveal any clinically significant endoscopic finding, we propose that patient selection for this invasive and costly procedure should be meticulous to avoid unnecessary financial burden on this deprived patient population. In this regard, we recommend that UGIE should be reserved mostly for patients with alarm symptoms. For all others, life-style modifications and anti-acid medications should first be tried before subjecting these individuals to endoscopic assessment.

### Authors’ Contribution:

**KM:** Conceived, designed and manuscript writing.

**FA:** Did statistical analysis and data interpretation final approval of manuscript.

**RB:** Helped in data collection and manuscript writing.

**AJ:** Did review and final approval of manuscript.

All authors are responsible and accountable for the accuracy and integrity of the work.
